# Supplementary material for: To what extent are the antimalarial markets in African countries ready for a transition to triple artemisinin-based combination therapies?
Source: PLoS One. 2021 Aug 31;16(8):e0256567. doi: 10.1371/journal.pone.0256567 (PMC8407563; doi:10.1371/journal.pone.0256567)
Supplement: S1 File — (ZIP) [file pone.0256567.s001.zip › Interview guides (ZIP)/1. Interview-NMCPs_Final_English.docx]

Interview Guide 1

**Project Title: Ethical, Social, Regulatory and Market related aspects of Deploying Triple Artemisinin-Based Combination Therapies for Malaria treatment in Africa: Case studies in Burkina Faso and Nigeria**

Target group-National Malaria Control Programmes

1. Introduction
   1. *Welcome the participant and briefly describe objectives of the project (explore sending information sheet prior to interview)*
   2. *Review Study Info Sheet & provide copy of Consent Form for signature*
   3. *Outline the format of interview (how long it will take)*
   4. *Allow time for questions and clarifications*
   5. *Ask permission to mention the affiliation (job function) of the respondent and audio-recording and start the recording*
2. Background of interviewee
   1. Could you please tell me a bit about yourself? i.e. your background and training?
   2. Could you tell a bit about the work of the NMCP and your role within the organization?
   3. Could you please elaborate on some of the key malaria’s control strategies (preventions, curative etc) in this country?
3. General views on drug development and malaria control
   1. What are the current processes for deploying new anti-malarial drugs in this country? Please explain/could you elaborate

- What is the role of the government in these processes?
  1. Could you please describe the regulatory and market related requirements for introducing new drugs/health products on the market?
  2. Can you tell me something about change of policy from monotherapies to ACTs for malaria treatment? Which lessons can we learn for future transitions to a new first-line drug?

1. Views on key ethical and regulatory considerations on deployment of TACTs
   1. Could you please share your views on the deployment of new combinations of antimalarial drugs in this country?

- What are your views on limiting patients’ choice to just TACTs for malaria treatments to prevent resistance (for public health benefits)?
  1. What are your views on a potential change from ACTs to TACTs as a first line treatment for malaria in our country, given that ACTs are still effective in this country?
  2. Studies have shown the possibilities of slight increase in minor side effects such as nausea, vomiting) but could prevent antimalarial drug resistance. What are your views? (Probe for individual risks/discomforts vs public benefit)
  3. As we explained in the information sheet, TACTs are likely to be initially deployed alongside ACTs. If that happens, how will this influence the current activities of the National Malaria Control Programmes in this Country? (probe for priorities of the NMCP)

1. Community engagement and uptake of TACTs
   1. What are the key strategies that could facilitate the deployment of TACTs in this country? (Probe for experiences from previous drug implementation programs)
   - In what key ways should local communities be engaged in discussions on deployment of TACTs in this country?
   - Which key communities and stakeholders should be targeted in these engagement activities?
   1. How will the deployment of TACTs influence the health seeking behavior of patients and community members, given the slight increase in minor side effects for patients?
2. Views on barriers to TACT deployment
   1. How would implementation programs look like in case TACT is included in national guidelines? Would this differ between public / private sector?
   2. How would the process of switching to TACT relate to switching to another ACT?
   3. What would be considerations to include medicines in guidelines that do not improve patient outcomes but rather reduce risks of resistance (eg. TACT while ACT is still effective? In what key ways should these barriers be addressed?
   4. In your view, is there potential capacity for local production of TACTs in this Country?
   5. Since ACTs are still effective in Africa, how will the NMCP facilitate the co-implementation of the two treatment regimens for Malaria in this country?

1. Market positioning: Policy
   1. Policy change: What would be the key considerations and challenges for changing national treatment guidelines from ACT to TACT: a triple combination of *currently registered* anti-malarial drug compounds, such as Artemether + Lumefantrine + Piperaquine?

- How long should this process take?
  1. To what extent are WHO global guidelines followed in developing national policy?
- What clinical evidence would be required for including TACT as first-line treatment in national guidelines?
  1. The WHO recommended ACT deployment since 2001 (because of Chloroquine and SP resistance). Most African countries, including Nigeria, accepted and implemented ACT only years later. What caused this delay between WHO guidelines and national guidelines and how could this be prevented for TACT?
  2. Nigeria included ACT in their guidelines in 2004. ACTwatch data shows that ACT availability in Nigeria was still very low in 2009, especially in the private sector. This improved significantly in more recent years. Could you explain what caused this delayed implementation and what caused the improvements later on?
  3. What lessons for TACT can we learn from previous drug transitions and the policy change processes?
  4. Are there any other external stakeholders that are important in policy change? How could they be engaged?
  5. What would NMCP do if failure rates of the first-line ACT in the country would get above 10%?

1. Market positioning: Commercial considerations
   1. It is likely that TACT will be slightly more expensive than ACT. What would be acceptable retail prices for TACT? How should price relate to ACT?

- What pricing strategies should be adopted for the public and for the private sector?
  1. What type of anti-malarial reimbursement subsidies do exist in the country (public and private sector)? How would TACT fit within these subsidy policies?
  2. What activities should the government take to make the prescription of TACT more attractive?
  3. What other considerations regarding affordability should be made before TACT can become first-line anti-malarial?
  4. We expect the number of tablets to be similar to the existing ACT, but if there is an increase in the number of tablets, what numbers would be acceptable?
- Any other considerations for tablet size, taste, solubility etc?
  1. The addition of a third component may have some slight side effects. For example, adding a third drug can results in more patients vomiting within one hour of treatment (1 per 100 for ACT, versus 3 in 100 for TACT). Would this be acceptable?
- How would this be for other malaria like side-effects such as fatigue, dizziness, headache etc?
  1. Are there any long-term deals or arrangements with ACT manufacturers or traders? Could this be a barrier to a switch to TACT?
  2. Are there any other economic or commercial considerations to make?

1. Market positioning: Import and distribution (public & private sector)
   1. How are anti-malarial medicines imported and distributed in the country?
   - What would be challenges to switch medicine import and distribution to TACT?
   - How is this for the public versus private sector?

9.2 How are medicines distributed from central to district levels?

- - What would be challenges here to switch from ACT to TACT?
  - How is this for the public versus private sector?
  1. What forecasting and capacity issues should be considered for TACT? How could be dealt with this (public versus private sector)?
  2. Would it be possible to immediately scale up TACT procurement and distribution in case:
  - TACT becomes first-line anti-malarial drug in national guidelines?
  - Failure rates of ACT start to increase (eg. above 10%)

1. Market positioning: implementation
   1. What type of information campaign/ training (to doctors, pharmacists, village health workers etc.) would be required with the implementation of TACT?

- What resources do exist in the country to facilitate this?
  1. What type of information campaigns would be appropriate for informing patients/population?
- What resources are in place (advertisement, tv spots, radio, billboards)?
  1. To what extent do clinicians/retailers/prescribers comply to new anti-malarials in national malaria guidelines (probe for poor, fair, good, very good)?
  2. Are there market monitor activities to ensure compliance to treatment guidelines?
- How would TACT fit these routines?
  1. Are there any other market related issues you would like to bring up?

1. Recommendations
   1. Based on our discussions, what recommendations would you give for addressing key challenges and barriers to deploying TACTs in Africa?
   2. Is there anything that we haven’t covered that you’d like to mention?

*Thank you very much for your insightful inputs to this project*
