# Supplementary material for: To what extent are the antimalarial markets in African countries ready for a transition to triple artemisinin-based combination therapies?
Source: PLoS One. 2021 Aug 31;16(8):e0256567. doi: 10.1371/journal.pone.0256567 (PMC8407563; doi:10.1371/journal.pone.0256567)
Supplement: S1 File — (ZIP) [file pone.0256567.s001.zip › Interview guides (ZIP)/1. Interview-NMCPs_final_French.docx]

Guide d’Entretien 1

**Intitulé du Projet : Aspects éthiques, sociaux, réglementaires et commerciaux du déploiement des combinaisons thérapeutiques à base d’artémisinine pour le traitement du paludisme en Afrique: Études de cas au Burkina Faso et au Nigeria**

**Groupe Cible – Programmes Nationaux de Lutte contre le Paludisme &**

**Directions Régionales de la Santé**

1. Introduction
   1. *Accueillir le participant et lui faire une brève description des objectifs du projet*

*(S’assurer de l’envoi de la fiche d’information avant l’entretien)*

- 1. *Parcourir la fiche d’information et remettre une copie de la fiche de consentement pour signature*
  2. *Exposer sur les grandes lignes de format de l’interview (items, durée…)*
  3. *Consacrer du temps pour les questions et les éclaircissements*
  4. *Demander une autorisation pour mentionner l’affiliation (poste) de la personne et pour faire un enregistrement audio de l’entretien*
  5. *Commencer l’entretien (et l’enregistrement si le répondant est d’accord)*

1. Profil du participant à l’entretien
   1. Pouvez-vous nous parler de vous-même ? c.-à-d. de votre formation, parcours, nombre d’années au sein de l’organisation ?
   2. Pouvez-vous nous parler du rôle du PNLP (resp. DRS) et de votre rôle au sein de l’organisation ?
   3. Parlez-nous de certaines de stratégies principales de lutte contre le paludisme (en matière de préventions et soins etc.) de notre pays
2. Opinions générales sur l’élaboration des médicaments et la lutte contre le paludisme
   1. Quelles sont les processus actuels de déploiement des nouveaux produits de lutte contre le paludisme dans notre pays. Expliquez

- Quel est le rôle du gouvernement dans ces processus ?
  1. Pouvez-vous décrire les exigences réglementaires et commerciales relatives à l’introduction de nouveaux médicaments/ produits de santé sur le marché ?
  2. Pouvez-vous nous dire un mot sur le changement de protocole des monothérapies aux ACT en matière de traitement antipaludique ?

Quelles leçons pouvons-nous en tirer pour les transitions à venir vers un nouveau médicament de première ligne ?

1. Opinions sur les considérations éthiques et réglementaires sur le déploiement des TACT
   1. Pouvez-vous partager votre point de vue sur le déploiement des nouvelles combinaisons de médicaments antipaludiques dans notre pays ?

- Que pensez-vous de la limitation du choix des patients aux TACT seulement pour le traitement antipaludique afin de prévenir une résistance
  (En termes de santé publique)
  1. Que pensez-vous d’un changement potentiel des ACT aux TACT comme médicament antipaludique de première ligne dans notre pays, vu que les ACT sont encore efficaces dans ce pays ?
  2. Des études ont montré des possibilités d’une légère accentuation des effets secondaires mineurs (comme la nausée et le vomissement) mais qui pourraient prévenir une résistance aux médicaments antipaludiques. Qu’en pensez-vous ?

(Position par rapport au risques/malaises individuels VS avantages publics)

- 1. Comme nous l’avons expliqué dans la fiche, il se peut que les TACT soient déployés avec les ACT. Si c’est le cas, comment cela influencera-t-il les activités actuelles des PNLP (resp. DRS) dans ce pays ?

(Identification des priorités des PNLP (resp. DRS))

1. Engagement Communautaire et Intérêt pour les TACT
   1. Quelles sont les principales stratégies qui pourraient faciliter le déploiement des TACT dans notre pays ?

(Identification des expériences issues de la mise en œuvre de programmes antérieurs de déploiement de traitement)

- Comment devrait-on impliquer les communautés locales dans les discussions sur le déploiement des TACT dans notre pays ?
- Quels sont les acteurs principaux qui devraient être ciblés dans ces activités d’engagements ?
  1. Comment le déploiement des TACT influencera-t-il le comportement de recherche de santé des patients et des membres de la communauté, au regard de la légère augmentation des effets secondaires mineurs sur les patients ?

1. Points de vue sur les barrières au déploiement des TACT
   1. A quoi pourraient ressembler les programmes de mise en œuvre, si les TACT sont intégrés dans les directives nationales ? Y aurait-il une différence entre leur mise en œuvre dans le secteur public et dans celui privé ?
   2. Quel pourrait être le lien entre un passage aux TACT et un passage à un autre ACT ?
   3. Que seraient les considérations à prendre en compte pour inclure dans les directives de prise en charge, des médicaments qui n’améliorent pas les résultats du patient mais plutôt réduisent les risques de résistance (Exemple des TACT alors que les ACT sont encore efficaces) ?
      Par quelles actions clés devrait-on lever ces barrières ?
   4. Selon vous, notre pays est-il à mesure de produire les TACT au niveau local ?
   5. Comme les ACT sont toujours efficaces en Afrique, comment le PNLP (resp. DRS) facilitera-t-il le déploiement conjoint de ces deux schémas thérapeutiques contre le paludisme dans notre pays ?
2. Positionnement sur le Marché : Politiques
   1. Changement de politique : Quels seraient principales considérations et les principaux défis à prendre en compte pour changer les protocoles nationaux de traitement des ACT aux TACT ?

- Quelle serait la durée de ce processus ?
  1. A quel point les directives mondiales de l’OMS sont-elles prises en compte et respectées dans l’élaboration d’un protocole national ?
- Quelle serait la preuve clinique exigée pour prendre en compte les TACT comme un médicament de première ligne dans les protocoles nationaux ?
  1. Concernant les TACT, quelles leçons pouvons-nous apprendre des transitions antérieures de médicaments et des processus de changement de protocole ?
  2. Existe-t-il d’autres acteurs externes qui sont importants dans le changement de protocole ? Comment pourrait-on les impliquer ?
  3. Que ferait le PNLP (resp. DRS) au cas où les taux d’échec des ACT de première ligne du pays seraient au-delà de 10% ?

1. Positionnement du Marché : Considérations commerciales
   1. Quel serait selon vous, les prix au détail acceptable des TACT ? Comment serait ce prix par rapport aux ACT ? Quelles stratégies de détermination de prix devrait-on adopter pour le secteur public/privé ?
   2. Quel type de subventions et/ou de remboursement de traitement antipaludique il y a-t-il dans le pays (secteur publique / privé) ? Comment pourrait-on adapter les TACT à ces politiques de subvention ?
   3. Quelles sont les activités/dispositions que le gouvernement devrait entreprendre pour rendre la prescription des TACT plus attrayante ?
   4. Quelles autres considérations relatives à l’accessibilité devrait-on prendre en compte avant que les TACT ne puissent être un traitement antipaludique de première ligne ?
   5. Nous espérons que le nombre de comprimés soit similaire aux ACT actuels toutefois, au cas où le nombre serait plus élevé, quel nombre serait acceptable selon vous ?

- Y-a-t-il d’autres considérations concernant la taille, le goût et solubilité du comprimé à prendre en compte ?
  1. L’addition d’une troisième composante peut avoir des effets secondaires légers. Par exemple, entrainer plus de vomissements chez des patients dans l’heure suivant le traitement (1 pour 100 pour les ACT contre 3 sur 100 pour les TACT).

Cela serait-il acceptable selon vous ?

- Qu’en serait-il pour les autres effets secondaires des produits anti palustres comme la fatigue, les vertiges, les maux de tête etc ?
  1. Qu’en sera-t-il selon vous, des contrats de long terme et/ou des accords avec les fabricants et/ou les commerciaux des ACT ? Cela pourrait-il constituer une barrière à la transition vers les TACT ?
  2. Existe-t-il d’autres considérations économiques ou commerciales prendre en compte ?

1. Positionnement du marché : Importation et distribution
   1. Comment importe-t-on et distribue-t-on les produits antipaludiques dans notre pays ?

- Quels seraient les défis du changement des importations et de la distribution des ACT vers les TACT ?
- Qu’en serait-il pour le secteur public comparativement au secteur privé ?
  1. Comment les médicaments sont-ils distribués depuis le niveau central jusqu’au niveau des districts ?
- Que seraient les défis du changement des ACT aux TACT ?
  1. Quelles difficultés en matière de prévision et de capacité, à prendre en compte concernant le TACT ?

Comment pourrait-on y faire face (secteur public vs rapport au privé)

- 1. Pourrait-on immédiatement vulgariser l’achat et la distribution des TACT au cas où :
- Ils deviennent le médicament de première ligne contre le paludisme dans les directives nationales
- Les taux d’échec des ACT commencent à s’accroître (exemple au-delà de 10%).

1. Positionnement sur le Marché : Mise en œuvre
   1. De quels types de campagne d’information et/ou de formation (pour les médecins, pharmaciens, infirmiers, agents de santé communautaires…) aurait-on besoin dans la mise en œuvre des TACT

- Quelles sont les ressources disponibles dans le pays pour faciliter cela ?
  1. Quel type de campagne d’information serait adéquat pour informer les patients/la population ?
- Quelles sont les ressources matérielles disponibles pour cela ?
  (publicité, spots TV, radio, tableaux d’information) ?
  1. Dans quelle mesure, les cliniciens/détaillants/prescripteurs respectent-ils les directives nationales sur les nouveaux médicaments contre le paludisme ?

(Pas du tout, moyennement, assez bien, très bien)

- 1. Existe-t-il des activités de suivi pour s’assurer du respect des directives de traitement ?
- Comment les TACT s’intègreraient-ils à ces activités de routine ?
  1. Existe-t-il d’autres difficultés relatives au positionnement sur le marché que vous souhaiteriez souligner ?

1. Recommandations
   1. En se basant sur nos échanges, quelles recommandations feriez-vous pour relever les principaux défis et briser les barrières au déploiement des TACT en Afrique ?
   2. Existe-t-il des omissions de notre part mais que vous souhaiteriez mentionner ?

*Merci pour vos contributions éclairées au présent projet*
