# Supplementary material for: To what extent are the antimalarial markets in African countries ready for a transition to triple artemisinin-based combination therapies?
Source: PLoS One. 2021 Aug 31;16(8):e0256567. doi: 10.1371/journal.pone.0256567 (PMC8407563; doi:10.1371/journal.pone.0256567)
Supplement: S1 File — (ZIP) [file pone.0256567.s001.zip › Interview guides (ZIP)/2. Interview-Regulators_Final_English.docx]

Interview Guide 2

**Project Title: Ethical, Social, Regulatory and Market related aspects of Deploying Triple Artemisinin-Based Combination Therapies for Malaria treatment in Africa: Case studies in Burkina Faso and Nigeria**

Target group-Regulatory officials

1. Introduction
   1. *Welcome the participant and briefly describe objectives of the project*
   2. *Review Study Info Sheet & provide copy of Consent Form for signature*
   3. *Outline the format of interview (how long will it take)*
   4. *Allow time for questions and clarifications*
   5. *Ask permission to mention the affiliation (job function) of the respondent and audio-recording*
2. Background of interviewee
   1. Could you please tell me a bit about yourself? i.e. your background and training and years within the organization?
   2. What is your current role at the National Regulatory Authority (Probe for Food and Drugs Administration)?
3. General views on drug development and malaria control
   1. Could you describe the regulatory landscape within the country? How does the process of registering new medicines in the country occur?

- How long would the general review process for new medication take?
  1. Are there currently records of counterfeiting and substandard anti-malaria drugs in the market? Would this be a risk for TACTs?
  2. Are there key lessons in the regulatory process that we can learn from the transition from monotherapies to ACTs for malaria treatment?

1. Market positioning: process of registration & market authorization
   1. How would registration take place for *a triple combination* *of currently registered anti-malarial drug compounds* such as the ACT compounds Artemether + Lumefantrine + Piperaquine?
   2. What barriers could be expected during the registration/market authorization of TACT in the country? And how could these barriers be tackled?
   3. To what extent is national drug regulation compliant with the WHO recommendation and/or with regulation of surrounding countries?
   4. Would the regulatory system be ready for a transition from ACT to TACT? Why (not)?
   5. The WHO recommended ACT deployment since 2001 (because of Chloroquine and SP resistance). Most African countries, including Nigeria, accepted and implemented ACT only years later. What caused this delay between WHO guidelines and national guidelines and how could this be prevented for TACT?
   6. Are there other important considerations to be made for the market authorization of TACT?
2. Market positioning: clinical considerations
   1. What type of clinical evidence would be required for registration and market authorization of TACT? Would for example locally acquired evidence be required?
   2. What would the national regulatory office do if failure rates of the first-line ACT in the country would get above 10%?
   3. We expect the number of tablets to be similar to the existing ACT, but if there is an increase in the number of tablets, what would be acceptable to the agency and the general population?
   4. The addition of a third component may have some slight side effects. For example, adding a third drug can result in more patients vomiting within one hour of treatment (1 per 100 for ACT, versus 3 in 100 for TACT). Would this be acceptable to the agency and the general population?
   5. How would this be for other malaria like side-effects such as fatigue, dizziness, headache etc?
3. Market Positioning: Policy & Affordability
   1. In many countries, there have been delays in the deployment of ACTs. Nigeria included ACT in their guidelines in 2004. ACTwatch data shows that ACT availability in Nigeria was still very low in 2009, especially in the private sector. This improved significantly in more recent years. Could you explain what caused this delayed implementation and what caused the improvements later on?
   2. How could the implementation of TACT be improved compared to the previous implementation of ACT?
   3. What would be appropriate/acceptable retail prices for TACT? How would these have to relate to ACT? How would the prices relate between public/private sector outlets?

- What activities should the government take to make the prescription of TACT more attractive?
- Are there any regulatory or policy measures that could be used to specifically engage the private sector into a transition to TACT
  1. It is likely that TACT will be slightly more expensive than ACT. What other considerations regarding affordability should be made before TACT can become first-line anti-malarial?
  2. What would be the role of long-term deals or arrangements with ACT manufacturers or traders? Could this be a barrier to a switch to TACT?
  3. What type of anti-malarial drug reimbursement subsidies do exist in the country (public and private sector)? How would TACT fit within these subsidy policies? What considerations would be made to have new medicines such as TACT subsidized?
  4. Are there any other market related issues you would like to bring up with regard to introduction of TACT?

1. Views on key ethical considerations on deployment of TACTs
   1. Could you please share your views on the key ethical considerations that should inform the development and deployment of new drugs in this country?
   2. What are your views on a potential change from ACTs to TACTs as a first line treatment for malaria in your country?
   3. In what ways does/should regulatory authorities facilitate the rapid deployment of TACTs?
2. Views on barriers to TACT deployment
   1. How would policy change to TACT look like? How long should this process take?
   2. What are the potential regulatory barriers to deploying TACTs in this country, given that ACTs are still effective and currently the first line of treatment? (Probe for ethical and regulatory barriers)
   3. In what key ways should these barriers be addressed?
   4. Delays in policy change have been highlighted in the literature, what do you think accounts for these delays
   5. How can illegal drug sales, drug peddlers/hawkers and counterfeiting/substandard drug sales affect the deployment of TACTs?
   6. What key steps should be adopted to facilitate the deployment of TACTs in this Country?
   7. In your view, are there potential local capacity for local production of TACTs in this Country?
   8. Since ACTs are still effective in Africa, how will this facilitate the co-implementation of the two treatment regimens for Malaria in this country?
3. Stakeholder engagement and uptake of TACTs
   1. What are the key strategies that will facilitate the uptake of TACTs in Africa?
   2. Which national stakeholders should be involved in discussions that can facilitate the update of TACTs in this Country?
   3. In your experience, what are some of the stakeholder engagement activities that could be the most effective?
4. Recommendations
   1. Based on our discussions, what recommendations would you give for addressing the key challenges and barriers to deploying TACTs in Africa?
   2. Is there anything that we haven’t covered that you’d like to mention?

*Thank you very much for your insightful inputs to this project*
