# Supplementary material for: To what extent are the antimalarial markets in African countries ready for a transition to triple artemisinin-based combination therapies?
Source: PLoS One. 2021 Aug 31;16(8):e0256567. doi: 10.1371/journal.pone.0256567 (PMC8407563; doi:10.1371/journal.pone.0256567)
Supplement: S1 File — (ZIP) [file pone.0256567.s001.zip › Interview guides (ZIP)/2. Interview-Regulators_final_French.docx]

Guide d’Entretien 2

**Intitulé du Projet : Aspects éthiques, sociaux, réglementaires et commerciaux du déploiement des combinaisons thérapeutiques à base d’artémisinine pour le traitement du paludisme en Afrique: Études de cas au Burkina Faso et au Nigeria**

**Groupe Cible - Autorités de Régulations (DGPML / ANRP)**

1. Introduction
   1. *Accueillir le participant et lui faire une brève description des objectifs du projet*

*(S’assurer de l’envoi de la fiche d’information avant l’entretien)*

- 1. *Parcourir la fiche d’information et remettre une copie de la fiche de consentement pour signature*
  2. *Exposer sur les grandes lignes de format de l’interview (items, durée…)*
  3. *Consacrer du temps pour les questions et les éclaircissements*
  4. *Demander une autorisation pour mentionner l’affiliation (poste) de la personne et pour faire un enregistrement audio de l’entretien*
  5. *Commencer l’entretien (et l’enregistrement si le répondant est d’accord)*
  6. *(poste) de la personne et faire un enregistrement audio et commencer l’enregistrement*

1. Profil de la personne interviewée
   1. Pouvez-vous nous parler de vous-même ? c.-à-d. de votre formation, parcours et du nombre d’années au sein de l’organisation ?
   2. Pouvez-vous nous parler du rôle de la DGPML/ANRP et de votre rôle au sein de cette organisation ?

(Comprendre le fonctionnement de l’administration en charge des Médicaments)

1. Opinions générales sur la mise au point du médicament et la lutte contre le paludisme
   1. Pouvez-vous décrire le cadre réglementaire du médicament au sein de notre pays ?

- Quel est le processus d’enregistrement des nouveaux médicaments dans le pays ?
- Quelle est la durée de ce processus?
  1. Existe-t-il présentement des cas de médicaments antipaludiques de contrefaçon et non standards sur le marché ? Cela constituerait-t-il un risque pour les TACT ?
  2. Existe-t-il des leçons importantes que nous pouvons tirer du passage des monothérapies à aux ACT pour le traitement du paludisme ?

1. Positionnement sur le Marché : Processus d’enregistrement et d’autorisation de mise sur le Marché
   1. Comment procèderait-on à l’enregistrement d’une *triple combinaison de composantes de médicament antipaludique actuellement enregistrées ?*
   2. Quelles sont les barrières auxquelles pourrait-t-on s’attendre pendant l’enregistrement/l’autorisation de mise sur le marché des TACT dans le pays ?

Comment pourrait-on lever ces barrières ?

- 1. Dans quelle mesure la règlementation nationale sur le médicament est-elle en accord avec les recommandations de l’OMS et/ou les règlementations des pays voisins ?
  2. Le système de régulation serait-il prêt pour une transition des ACT aux TACT ?

Pourquoi (resp. Pourquoi pas) ?

- 1. Existe-t-il d’autres considérations importantes concernant l’autorisation des TACT sur le marché qui devraient être prises en compte ?

1. Positionnement sur le Marché : Aspects cliniques
   1. Quels types de preuve clinique sont nécessaires pour l’enregistrement et l’autorisation de mise sur le marché des TACT ?

Quelle serait par exemple, la preuve obligatoire au niveau local ?

- 1. Que ferait le Bureau de régulations au cas où les taux d’échec des ACT de première ligne excèderaient 10% dans notre pays ?
  2. Nous espérons que le nombre de comprimés soit similaire à celui des ACT, toutefois au cas où ce nombre augmenterait, quel serait le nombre acceptable pour la DGPML/ANRP et pour la population en générale selon vous ?
  3. L’ajout d’une troisième composante peut entrainer une légère accentuation des effets secondaires. Par exemple, entrainer plus de vomissements chez des patients dans l’heure suivant le traitement (1 sur 100 pour les ACT contre 3 sur 100 pour les TACT). Ce taux serait-il acceptable pour la DGPML/ANRP et la population en général, selon vous?
  4. Qu’en serait-il pour les autres effets secondaires de médicament anti palustres comme la fatigue, les vertiges, les maux de tête etc. ?

1. Positionnement sur le Marché : Politique et Accessibilité
   1. Dans plusieurs pays, il y a eu des retards dans le déploiement des ACT. Cela a-t-il été également le cas dans votre pays ?

Pouvez-vous donner les facteurs explicatifs du retard (resp.de la rapidité) de ce déploiement ?

- 1. Quel serait selon vous, les prix au détail acceptable des TACT ? Comment serait ce prix par rapport aux ACT ? Quelles stratégies de détermination de prix devrait-on adopter pour le secteur public/privé ?
- Quelles sont les activités/dispositions que le gouvernement devrait entreprendre pour rendre la prescription des TACT plus attrayante ?
- Existe-t-il des mesures politiques ou règlementaires qui pourraient être utilisées pour impliquer le secteur privé de manière spécifique dans une transition vers les TACT ?
  1. Quelles autres considérations sur l’accessibilité devrait-on prendre en compte avant que les TACT ne puissent être un médicament antipaludique de première ligne ?
  2. Qu’en sera-t-il selon vous, des contrats de long terme et/ou des accords avec les fabricants et/ou les commerciaux des ACT ? Cela pourrait-il constituer une barrière à la transition vers les TACT ?
  3. Quel type de subventions et/ou de remboursement de médicament contre le paludisme existe-t-il dans le pays (secteur publique et privé) ? Comment pourrait-on adapter les TACT à ces politiques de subvention ?
  4. Existe-t-il d’autres difficultés relatives au marché que vous souhaiteriez soulever concernant l’introduction de TACT ?

1. Opinions sur les considérations éthiques sur le déploiement des TACT
   1. Quelles sont selon vous, les principales considérations éthiques à prendre en compte dans notre pays pour l’élaboration et de déploiement de nouveaux médicaments ?
   2. Que pensez-vous d’un changement potentiel des ACT aux TACT comme traitement antipaludique de première ligne dans votre pays ?
   3. Comment les autorités de régulations devraient/peuvent-elles faciliter le déploiement rapide des TACT ?
2. Points de vue sur les barrières au déploiement des TACT
   1. Comment se ferait selon vous un changement de politique des ACT aux TACT ? Quelle serait la durée de ce processus ?
   2. Quelles sont les barrières règlementaires potentielles au déploiement des TACT dans notre pays, étant que donné que les ACT sont toujours efficaces et constituent présentement le traitement de première de ligne ?

(Barrières éthiques et règlementaires)

- 1. Comment devrait-on briser ces barrières ?
  2. Les retards du changement de politique ont été mis en exergue dans la littérature, selon vous qu’est ce qui justifie ces retards ?
  3. Comment les ventes illégales de médicaments, les ventes de médicament de contrefaçon et non conforme aux règles…peuvent-elles affecter le déploiement des TACT ?
  4. Quelles sont les principales étapes que l’on devrait adopter pour faciliter le déploiement des TACT dans notre pays ?
  5. Selon vous, notre pays possède-t-il une potentialité pour une production locale des TACT ?
  6. Comme les ACT sont toujours efficaces en Afrique, comment cette situation facilitera-t-elle la mise en œuvre conjointe de deux régimes de traitement pour le paludisme dans ce pays ?

1. Engagement des Acteurs et l’adoption pour les TACT
   1. Quelles sont les stratégies clés qui pourront faciliter l’adoption des TACT en Afrique ?
   2. Quels sont acteurs nationaux que l’on devrait impliquer dans les échanges car ils peuvent faciliter l’adoption des TACT dans notre pays ?
   3. Selon votre expérience, citez quelques activités d’engagement des acteurs qui pourraient être les plus efficaces.
2. Recommandations
   1. En se basant sur nos échanges, quelles recommandations feriez-vous pour relever les principaux défis et briser les barrières au déploiement des TACT en Afrique ?
   2. Existe-t-il des omissions de notre part mais que vous souhaiteriez mentionner ?

*Merci pour vos contributions éclairées au présent projet*
