# Supplementary material for: To what extent are the antimalarial markets in African countries ready for a transition to triple artemisinin-based combination therapies?
Source: PLoS One. 2021 Aug 31;16(8):e0256567. doi: 10.1371/journal.pone.0256567 (PMC8407563; doi:10.1371/journal.pone.0256567)
Supplement: S1 File — (ZIP) [file pone.0256567.s001.zip › Interview guides (ZIP)/3. Interview-Distributors_Final_English.docx]

Interview Guide 3

**Project Title: Ethical, Social, Regulatory and Market related aspects of Deploying Triple Artemisinin-Based Combination Therapies for Malaria treatment in Africa: Case studies in Burkina Faso and Nigeria**

Target group-Distributors and Traders

Individual Interviews

1. Introduction
   1. *Welcome the participant and briefly describe objectives of the project*
   2. *Review Study Info Sheet & provide copy of Consent Form for signature*
   3. *Outline the format of interview (how long will it take)*
   4. *Ask permission to mention the affiliation (job function) of the respondent and audio-recording*
2. Background of interviewee
   1. Could you please tell me a bit about yourself? i.e. your background and training and years of experience within the supply chain?
   2. Are you providing drugs to the public (governmental) sector and/or the private (commercial) sector?
   3. Could you describe the anti-malarial distribution chain? (from manufacturers to patients) What your role is in this process?
   4. How does the import and distribution of anti-malarials differ between the public and the private sector?
3. General views on drug development and malaria control
   1. Could you describe the process in which distribution chains for ACT were established? How did ACT distribution differ from monotherapies, e.g. chloroquine?
   2. What would be suitable implementation programs in case TACT would be included in national guidelines? How would this differ between public / private sector?

- What barriers could be expected for implementing TACT in the national distribution chain?
  1. How would you describe the current regulatory environment for anti-malarial drugs in your country. How would this affect the market positioning of TACT?
  2. What measures should the government take to address potential ACT resistance (probe for knowledge of development of TACTs)
  3. Are there currently counterfeiting and substandard anti-malarial drugs in the market? Would this be a risk for TACT?

**Market Positioning**

1. Market positioning: Commercial considerations
   1. It is likely that TACT will be slightly more expensive than ACT. What would be appropriate/acceptable retail/market prices for TACT? How would these have to relate current price to ACT? How would the prices relate between public/private sector outlets?

- What activities should the government take to make the prescription of TACT more attractive?
  1. What would be acceptable manufacturer prices for you to start acquiring and/or stock TACT instead of ACT? How would these have to relate current price to ACT?
  2. How would a transition to TACT affect your business and that of other drug distributors/ traders?
  3. Are there any long-term deals or arrangements with ACT manufacturers or traders? Could this be a barrier to a switch to TACT?
  4. Are there any other economic or commercial considerations that that should be taken in consideration for the distribution of TACT?

1. Market positioning: drug delivery
   1. Nigeria included ACT in their guidelines in 2004. ACTwatch data shows that ACT availability in Nigeria was still very low in 2009, especially in the private sector. This improved significantly in more recent years. Could you explain what caused this delayed implementation and what caused the improvements later on?
   2. You explained how anti-malarial drugs are procured and imported from manufacturer into the borders of the country? What would be the challenges to switch this from ACT to TACT? How is this for the public versus private sector?
   3. How are the drugs then distributed to the district/village level? What would be the challenges to switch this from ACT to TACT? How is this for the public versus private sector?
   4. What would be challenges for integrating TACTs in the public and private sector distribution chains?
   5. How is this for public and private sector?
   6. Are their subsidy regimes for anti-malaria drugs in this country? If yes who are the partners/collaborators involved in the subsidy arrangements?
   7. What lessons could we learn from previous drug transition in the process of switching to another drug? How can we translate these lessons to TACT?
   8. Are there any other commercial or market related issues that you think is relevant for a transition to TACT?
2. Market positioning: Attitudes to TACT
   1. Would you and other drug distributors/traders be aware of risk of drug resistance? How would this affect their attitude towards TACT?
   2. How is decided what medicines you stock (e.g. price, guidelines, patient demand, availability)? How would risk of resistance affect your decision? How is this for public versus private sector?

- To what extent do national guidelines affect your stocking decisions?
  1. When would distributors/traders be willing to switch to TACT? How is this for public versus private sector?
  2. How long would it take to phase out ACT from distribution chains? What would be challenges to do so?

1. Market positioning: Storage/forecasting
   1. How are anti-malarials in the country stored in public and private sector?

- Would there be any storage conditions (for example temperature and humidity) that needs to be considered for TACT?
  1. Are there any other forecasting or capacity issues you would like to bring up for the transition to TACT? (public versus private sector)?
  2. Would it be possible to immediately scale up TACT procurement and distribution in case:
- TACT becomes first-line anti-malarial drug in national guidelines?
- Failure rates of ACT start to increase

**Ethics**

1. Views on barriers to TACT deployment
   1. What are other potential barriers to distribution of TACTs in this country, given that ACTs are still effective and currently the first line of treatment? (Probe for ethical and regulatory barriers)
   2. In what key ways should these barriers be addressed?
   3. What key steps should be adopted to facilitate the deployment of TACTs in this Country
   4. In your view, are there potential local capacity for local production of TACTs in this Country?
   5. Are there any other market related issues you would like to bring up?
   6. Is the market in your country ready for a transition from ACT to TACT?
2. Recommendations
   1. Based on our discussions, what recommendations would you give for addressing the key challenges and barriers to deploying TACTs in Africa?
   2. Is there anything that we haven’t covered that you’d like to mention?

*Thank you very much for your insightful inputs to this project*
