# Supplementary material for: To what extent are the antimalarial markets in African countries ready for a transition to triple artemisinin-based combination therapies?
Source: PLoS One. 2021 Aug 31;16(8):e0256567. doi: 10.1371/journal.pone.0256567 (PMC8407563; doi:10.1371/journal.pone.0256567)
Supplement: S1 File — (ZIP) [file pone.0256567.s001.zip › Interview guides (ZIP)/3. Interview-Distributors_final_French.docx]

Guide d’Entretien 3

**Intitulé du Projet : Aspects éthiques, sociaux, réglementaires et commerciaux du déploiement des combinaisons thérapeutiques à base d’artémisinine pour le traitement du paludisme en Afrique: Études de cas au Burkina Faso et au Nigeria**

**Groupe cible – Distributeurs / Fournisseurs**

1. Introduction
   1. *Accueillir le participant et lui faire une brève description des objectifs du projet*

*(S’assurer de l’envoi de la fiche d’information avant l’entretien)*

- 1. *Parcourir la fiche d’information et remettre une copie de la fiche de consentement pour signature*
  2. *Exposer sur les grandes lignes de format de l’interview (items, durée…)*
  3. *Consacrer du temps pour les questions et les éclaircissements*
  4. *Demander une autorisation pour mentionner l’affiliation (poste) de la personne et pour faire un enregistrement audio de l’entretien*
  5. *Commencer l’entretien (et l’enregistrement si le répondant est d’accord)*

1. Profil de la personne interviewée
   1. Pouvez-vous nous parler de vous-même ? c.-à-d. de votre parcours, formation et du nombre d’années d’expérience dans la chaîne d’approvisionnement ?
   2. Etes-vous un fournisseur des médicaments au secteur public (gouvernement) et/ou du secteur privé (commercial) ?
   3. Pouvez-vous faire une description de la chaîne de distribution des médicaments antipaludiques (depuis les industries jusqu’aux patients) ? Quel est votre rôle dans ce processus ?
   4. Quelle est la différence entre le secteur public et le privé en matière d’importation et de distribution des produits antipaludiques ?
2. Opinions générales sur le développement du médicament et la lutte contre le paludisme
   1. Pouvez-vous décrire le processus par lequel les chaînes de distribution des ACT ont été définies ? En quoi la distribution des ACT a été différente des antis palustre mono thérapeutique comme la chloroquine ?
   2. Quel serait, selon vous, le processus dans le au cas où les TACT seraient inclus dans les directives nationales ? Quelle serait la différence entre secteur public et le privé ?

- Quelles pourraient être les barrières auxquelles il faut s’attendre dans la mise en œuvre des TACT dans la chaîne de distribution ?
  1. Quelle description feriez-vous du cadre réglementaire actuel des médicaments antipaludiques dans votre pays ? Comment cela affecterait-il le positionnement des TACT sur le marché ?
  2. Quelles sont les mesures que le gouvernement devrait prendre pour faire face à la potentielle résistance des ACT ?
     (connaissance sur le développement des TACT)
  3. Existe-t-il présentement des anti palustres contrefaits et/ou non standards sur le marché ? Leur existence constituerait-elle une menace pour les TACT ?

**Positionnement sur le Marché**

1. Positionnement sur le marché: Considérations commerciales
   1. Quels seraient selon vous, les prix de vente au détail/en gros, adéquats/acceptables pour les TACT? Quel serait le lien entre ces prix et ceux des ACT ? Que seraient les prix entre les débouchés du secteur public/ privé ?

- Quelles sont les activités/dispositions que le gouvernement devrait entreprendre afin de rendre la prescription des TACT plus attrayante ?
  1. Pour vous distributeurs, quels seraient les prix acceptables pour commencer à faire un stock des TACT au lieu des ACT ? Que serait le lien entre ces prix et les prix actuels des ACT ?
  2. En quoi la transition vers les TACT affecterait-elle l’activité des distributeurs ?
  3. Qu’en sera-t-il selon vous, des contrats de long terme et/ou des accords avec les fabricants et/ou les commerciaux des ACT ? Cela pourrait-il être une barrière à la transition vers les TACT ?
  4. Existe-t-il autres considérations économiques or commerciales que l’on devrait prendre en compte pour la distribution des TACT ?

1. Positionnement sur le marché: Livraison du médicament
   1. Vous nous avez expliqué le processus selon lequel les médicaments anti palustres sont importés depuis les fabricants jusqu’aux frontières du pays? Quels seraient les défis du changement des ACT aux TACT ?

- En termes de secteur public par rapport au privé
  1. Comment les médicaments sont-ils ensuite distribués jusqu’au district/village ? Quels seraient les défis dans ce circuit lors du changement des ACT aux TACT ? Quelle serait la différence entre le secteur public et celui privé ?
  2. Quels seraient les difficultés pour intégrer les TACT dans les chaînes de distributions? Quelle serait la différence entre le secteur public et celui privé ?
  3. Existe-t-il des régimes de subventions pour les médicaments contre le paludisme ? Si oui, quels sont les partenaires/collaborateurs impliqués dans les dispositions de la subvention ?
  4. Quelles leçons pouvons-nous tirer d’une transition antérieure ayant impliqué un basculement collectif vers un autre médicament ? Comment pouvons transposer ces leçons aux TACT ?
  5. Existe-t-il d’autres considérations économiques ou commerciales à prendre en compte  à prendre en compte dans le cadre de la transition vers les TACT ?

1. Positionnement sur le marché: Attitudes envers le TACT
   1. Les distributeurs/vendeurs de médicament sont-ils conscients des risques de résistance aux médicaments (= pharmaco résistance)?

Comment cela peut-il influencer leurs attitudes vers les TACT ?

- 1. Comment décidez-vous le choix des médicaments que vous mettez en stock (ex critères de prix, directives, demande des patients, disponibilité…) ?

Comment intégrer-vous le risque de résistance dans votre décision ?
Qu’en est-il du secteur public par rapport à celui privé ?
Quel est le rôle des directives nationales en matière de décisions de stockage ?

- 1. A quel moment, les distributeurs/vendeurs seraient-il prêts à migrer vers les TACT ? Qu’en est-il du secteur public comparé à celui privé ?
  2. Quelle durée pourrait-on prendre pour retirer les ACT des chaînes de distribution ? Quels seraient les défis?

1. Positionnement sur le marché: Stockage/ prévision
   1. Comment stocke-t-on les médicaments anti palustres du pays dans le secteur public et dans celui privé ?

- Existerait-il des conditions de stockage (par exemple température, humidité…) qui il faut prendre en compte concernant les TACT ?
  1. Existe-t-il d’autres difficultés de prévision ou stockage que vous aimeriez soulever pour la transition vers le TACT? (secteur public comparé au privé)
  2. Serait-il immédiatement possible de vulgariser l’approvisionnement et la distribution du TACT au cas où
- Les TACT deviennent un médicament de première ligne pour la lutte contre le paludisme dans les protocoles nationaux
- Les taux d’échec des ACT commencent à augmenter

**Ethique**

1. Opinions sur les barrières au déploiement des TACT
   1. Quelles sont les barrières potentielles à la distribution des TACT dans notre pays vu que les ACT sont toujours efficaces et constituent le traitement de première ligne ? (les barrières éthiques et règlementaires)
   2. Comment pourrait-on lever ces barrières?
   3. Quelles sont les principales étapes à adopter pour faciliter le déploiement des TACT dans notre pays ?
   4. Selon vous, existe-t-il des capacités locales potentielles pour les produire localement les TACT ?
   5. Existe-t-il d’autres défis du marché que vous aimeriez souligner ?
   6. Dans notre pays, le marché est-il prêt pour la transition des ACT au TACT ?
2. Recommandations
   1. En se basant sur nos échanges, quelles recommandations feriez pour relever les principaux défis et briser les barrières au déploiement des TACT en Afrique
   2. Existe-t-il des omissions de notre part mais que vous souhaiteriez mentionner ?

*Merci pour vos contributions éclairées au présent projet*
