# Supplementary material for: To what extent are the antimalarial markets in African countries ready for a transition to triple artemisinin-based combination therapies?
Source: PLoS One. 2021 Aug 31;16(8):e0256567. doi: 10.1371/journal.pone.0256567 (PMC8407563; doi:10.1371/journal.pone.0256567)
Supplement: S1 File — (ZIP) [file pone.0256567.s001.zip › Interview guides (ZIP)/4. Interview-Clinicians _Final_English.docx]

Interview Guide 4

**Project Title: Ethical, Social, Regulatory and Market related aspects of Deploying Triple Artemisinin-Based Combination Therapies for Malaria treatment in Africa: Case studies in Burkina Faso and Nigeria**

Target group-Clinicians/Prescribers/Pharmacists/Drug store employees

Individual Interviews

1. Introduction
   1. *Welcome the participant and briefly describe objectives of the project*
   2. *Review Study Info Sheet & provide copy of Consent Form for signature*
   3. *Outline the format of interview (how long it will take)*
   4. *Allow time for questions and clarifications*
   5. *Ask permission to mention the affiliation (job function) of the respondent and audio-recording*
   6. *Ask for permission to audio-record and start the recording*
2. Background of interviewee
   1. Could you please tell me a bit about yourself? i.e. your background and training and the years of work experience?
   2. Could you tell me a bit about the hospital/clinic/pharmacy you work?
   3. Are you working within the public (governmental) sector and/or in the private (commercial) sector?
   4. To what extent does the anti-malarial prescription behavior of clinicians/prescribers differ between public and private sector?
3. General views on drug development and malaria control
   1. Are you familiar with the antimalarial treatment guidelines and policy in your country?

- Could you please elaborate on how current guidelines influence your prescription behavior?
- What difficulties do you experience in applying guidelines for malaria treatment in your country?
  1. What measures should the government take to address the risk of ACT resistance (probe for knowledge of development of TACTs)
  2. How would implementation programs look like in case TACT is included in national guidelines? How would this differ between public / private sector?
  3. From your experience how satisfied are the clients to the current ACTs? (probe for cost, dose formulation, frequency and durations of use)

1. Views on key ethical and regulatory considerations on deployment of TACTs
   1. Could you please share your views on the deployment of new combinations of antimalarial drugs in this country?

- What are your views on a potential change from ACTs to TACTs as a first line treatment for malaria in our country, given that ACTs are still effective in this country?
- What are your views on limiting patients’ choice to just TACTs for malaria treatments to prevent resistance (for public health benefits)?
  1. Studies have shown the possibilities of slight increase in minor side effects such as nausea, vomiting) but could prevent antimalarial drug resistance. What are your views? (Probe for individual risks/discomforts vs public benefit)

1. Community engagement and uptake of TACTs
   1. What are the key strategies that could facilitate the uptake of TACTs in your country?
   2. In what key ways should local communities be engaged in discussions on deployment of TACTs in your country?
   3. How do you think the deployment of TACTs would affect the health seeking behavior of patients and community members?
   4. What type (strategies) of community and public engagement is necessary prior to and during deployment?

- Which key communities and stakeholders should be targeted in these engagement activities?

1. Views on barriers to TACT deployment
   1. What are the potential barriers to deploying TACTs in your country, given that ACTs are still effective and currently the first line of treatment? (Probe for ethical and regulatory barriers)
   2. In what ways should these barriers be addressed?
2. Market positioning: Commercial considerations
   1. What commercial considerations would clinicians and pharmacists have to take in stocking TACT and prescribing them to patients? How would this be in the public versus the private sector?

- How could a transition to TACT affect their business or profitability?
- What different considerations should be made for the public versus private sector?
  1. What are common retail prices for currently used ACT within the country? What would be acceptable retail prices for TACT in public/private sector outlets? It is likely that TACT will be slightly more expensive than ACT. How would the retail prices of TACT have to relate to prices of ACT?
- What activities should the government take to make the prescription of TACT more attractive compared to ACT?
  1. What other considerations regarding prices and affordability should be made before TACT can become first-line anti-malarial?
- What other factors influence the selection of appropriate anti-malarial treatment (e.g price, guidelines, marketing, informal advice by colleagues, demand or requirements of consumers/ end-users)
  1. What would be the role of medical delegates of the industry who market products to prescribers, pharmacists etc.

1. Market positioning: implementation
   1. What type of training/ information should be provided to inform prescribers and clinicians about a switch to TACT?

- How would this be in the public versus private sector
  1. How would the local marketing strategies of the pharmaceutical companies affect your decision to switch to TACT?
  2. What other implementation considerations should be made for a transition to TACT?

1. Market positioning: Stocking
   1. Are clinicians and pharmacists (in public and private sector) generally knowledgeable about risks of resistance? How would this affect their attitude towards TACT?
   2. How you decide what medicines are being stocked in your facility (e.g. price, guidelines, patient demand, availability)?

- If risk of resistance not emerged among the criteria, ask if it can be one of them
- How would risk of resistance be part of your decision to switch to TACT?
  1. How would inclusion of TACT in national guidelines affect the adoption of TACT by clinicians and prescribers?
- How would this be in public versus the private sector

1. Market positioning: Prescription to patients
   1. We expect the number of tablets to be similar to the existing ACT, but if there is an increase in the number of tablets, what would be acceptable?
   2. The addition of a third component may have some slight side effects. For example, adding a third drug can results in more patients vomiting within one hour of treatment (1 per 100 for ACT, versus 3 in 100 for TACT). Would this be acceptable?

- How would this be for other side-effects such as fatigue, dizziness, headache etc., that might slightly increase with TACT compared to ACT?
  1. What considerations should be made for TACT related to different pills or doses for children and adults?
  2. Are there any other promotion / packaging / blistering or other considerations that should be made for TACT?
  3. What considerations should be made for informing patients about TACT?

1. Recommendations
   1. Based on our discussions, what recommendations would you give for addressing the key challenges and barriers to deploying TACTs in your country and in Africa?
   2. Is there anything that we haven’t covered that you’d like to mention?

*Thank you very much for your insightful inputs to this project*
