# Supplementary material for: To what extent are the antimalarial markets in African countries ready for a transition to triple artemisinin-based combination therapies?
Source: PLoS One. 2021 Aug 31;16(8):e0256567. doi: 10.1371/journal.pone.0256567 (PMC8407563; doi:10.1371/journal.pone.0256567)
Supplement: S1 File — (ZIP) [file pone.0256567.s001.zip › Interview guides (ZIP)/4. Interview-Clinicians_final_French.docx]

Guide d’Entretien 4

**Intitulé du Projet : Aspects éthiques, sociaux, réglementaires et commerciaux du déploiement des combinaisons thérapeutiques à base d’artémisinine pour le traitement du paludisme en Afrique: Études de cas au Burkina Faso et au Nigeria**

**Groupe cible : Cliniciens/Prescripteurs/Pharmaciens/Employés des pharmacies**

*Entretiens Individuels*

1. Introduction
   1. *Accueillir le participant et lui faire une brève description des objectifs du projet*

*(S’assurer de l’envoi de la fiche d’information avant l’entretien)*

- 1. *Parcourir la fiche d’information et remettre une copie de la fiche de consentement pour signature*
  2. *Exposer sur les grandes lignes de format de l’interview (items, durée…)*
  3. *Consacrer du temps pour les questions et les éclaircissements*
  4. *Demander une autorisation pour mentionner l’affiliation (poste) de la personne et pour faire un enregistrement audio de l’entretien*
  5. *Commencer l’entretien (et l’enregistrement si le répondant est d’accord)*

1. Profil de la personne interviewée
   1. Pouvez-vous nous parler de vous-même ? c.-à-d. de votre formation, parcours et du nombre d’années d’expérience professionnelle ?
   2. Pouvez-vous nous parler de l’hôpital/ de la clinique/ de la pharmacie où vous travaillez ?
   3. Travaillez-vous dans le secteur public (gouvernement) et/ou dans le secteur privé (commercial) ?
   4. Dans quelle mesure le comportement pour les prescriptions anti palustres des cliniciens /prescripteurs diffère-t-il entre le secteur public et privé ?
2. Points de vus généraux sur le développement des médicaments et la lutte contre le paludisme
   1. Etes-vous familier avec les directives/le protocole de traitement antipaludique de notre pays ?

- Pouvez-vous parler de la manière dont les directives influencent votre comportement de prescription ?
- Quelles sont les difficultés que vous rencontrez dans la mise en œuvre des directives sur les traitements antipaludiques dans notre pays ?
  1. Quelles sont les mesures que le gouvernement devrait prendre pour faire face à la résistance aux ACT ? (= connaissance sur le développement des TACT)
  2. A quoi pourraient ressembler les programmes de mise en œuvre, si les TACT sont intégrés dans les directives nationales ?

Y aurait-il une différence entre leur mise en œuvre dans le secteur public et dans celui privé ?

- 1. Selon votre expérience quel est le niveau de satisfaction des clients sur les ACT actuels ?

(En termes de coût, formulation de la dose, fréquence et durée d’utilisation…)

1. Points de vue sur les considérations éthiques et règlementaires sur le déploiement des TACT
   1. Pouvez-vous partager votre point de vue sur le déploiement des nouvelles combinaisons de médicaments anti palustres dans votre pays ?

- Que pensez-vous d’un changement potentiel des ACT aux TACT comme un traitement antipaludique de première ligne dans votre pays vu que les ACT sont encore efficaces dans ce pays ?
- Que pensez-vous de la limitation du choix des traitements antipaludiques des patients aux TACT uniquement afin de prévenir la résistance (pour des raisons de santé publique) ?
  1. Des études ont montré des possibilités d’une augmentation légère des effets secondaires (comme la nausée, le vomissement..) avec l’utilisation des TACT, mais cela pourrai prévenir la résistance aux antipaludiques actuellement utilises (ACT). Qu’en pensez-vous ?

(Position par rapport au risques/malaises individuels VS avantages publics)

1. Engagement communautaire et Engouement pour les TACT
   1. Quelles sont, selon vous, les principales stratégies qui pourraient faciliter l’adoption des TACT dans notre pays ?
   2. Comment devrait-on impliquer les communautés locales dans la discussion relative au déploiement des TACT dans notre pays ?
   3. Selon vous, comment le déploiement des TACT influencera-t-il le comportement de recherche de la santé du patient et des membres de la communauté ?
   4. Quel type d’engagement communautaire et public (stratégie) est nécessaire avant et pendant le déploiement ?

- Quelles sont les communautés et acteurs principaux que l’on devrait cibler dans les activités d’engagement ?

1. Points de vue sur les barrières au déploiement des TACT
   1. Quelles sont les barrières potentielles au déploiement des TACT dans notre pays, vu que les ACT sont toujours efficaces et constituent un traitement de première ligne ?
      (= les barrières éthiques et règlementaires)
   2. Comment devrait-on lever ces barrières ?
2. Positionnement sur le marché : considérations commerciales
   1. Quelles sont les considérations commerciales que les cliniciens et les pharmaciens devraient prendre en compte lors du stockage des TACT et de leur prescription aux patients ?

- Comment une transition vers les TACT affectera-t-elle leurs affaires ou leur rentabilité ?
- Quelles sont les différentes considérations que l’on devrait prendre en compte pour le secteur public par rapport à celui privé ?
  1. Quels sont les prix au détail moyens pour les ACT actuellement utilisés dans le pays ?

Quels seraient les prix au détail acceptables des TACT, sur les marchés du secteur public et sur ceux du secteur privé ? Quels seraient les liens entre ces prix et ceux des ACT ?

- Quelles sont les activités que le gouvernement devrait entreprendre pour rendre la prescription des TACT plus attractive par rapport aux ACT ?
  1. Quelles sont les considérations concernant l’accessibilité que l’on devrait avoir avant que les TACT deviennent un traitement antipaludique de première ligne ?
  2. Quels sont les autres facteurs qui influencent le choix d’un traitement antipaludique adéquat ?

(ex prix, directives, marketing, conseils informels des collègues…)

- Qu’en est-il des délégués médicaux qui présentent les produits auprès des prescripteurs, des pharmaciens… ? (En retour, ils ont des gains)
- Qu’en est-il des demandes ou exigences des consommateurs/ utilisateurs finaux ?

1. Positionnement sur le marché : mise en œuvre
   1. Quel type de formation / d’information devrait-on fournir afin d’informer les prescripteurs et les cliniciens du changement pour les TACT ?

- Qu’en serait-il dans le secteur public par rapport au privé ?
  1. Comment les stratégies locales de marketing des firmes pharmaceutiques affecteront-elles votre décision de changement pour les TACT ?
  2. Quelles autres considérations de mise en œuvre devrait-on avoir pour la transition vers les TACT ?

1. Positionnement sur le marché : Stockage
   1. Les cliniciens et les pharmaciens (du secteur public et privé) sont-ils généralement informés des risques de résistance ?

Quel effet cela aurait-il sur leur attitude vers les TACT ?

- 1. Selon quels critères faites-vous le choix des médicaments à stocker dans votre structure ?
     (ex prix, directives, demande du patient, accessibilité…)
- Si le risque de résistance n’est pas parmi des critères, demandez s’il peut être l’un d’eux
- Comment le risque de résistance serait-il pris en compte dans votre décision de changement pour les TACT ?
  1. Comment l’inclusion des TACT dans les protocoles nationaux affecterait-elle l’adoption des TACT par les cliniciens et prescripteurs ?
- Qu’en serait-il dans le secteur public par rapport à celui privé ?

1. Positionnement sur le marché : Prescription aux patients
   1. Nous espérons que le nombre de comprimés soit similaire à celui des ACT, toutefois au cas où ce nombre augmenterait, quel serait le nombre acceptable selon vous ?
   2. L’ajout d’une troisième composante peut entrainer une légère accentuation des effets secondaires. Par exemple, entrainer plus de vomissements chez des patients dans l’heure suivant le traitement (1 sur 100 pour l’ACT contre 3 sur 100 pour le TACT).
      Ce taux serait-il acceptable, selon vous ?

- Qu’en serait-il pour les autres effets secondaires comme la fatigue, les vertiges, les maux de tête etc. qui pourraient s’accroître légèrement avec les TACT par rapport aux ACT ?
  1. Quelles seraient les considérations relatives aux différents comprimés ou doses pour enfants et adultes à prendre en compte concernant les TACT ?
  2. Existe-t-il d’autres aspects (promotion / emballage / blister…) ou autres considérations sur les TACT dont on devrait tenir compte ?
  3. Quelles sont les considérations qui devraient être prises en compte pour informer les patients par rapport au TACT ?

1. Recommandations
   1. En se basant sur nos échanges, quelles recommandations feriez-vous pour relever les principaux défis et les barrières au déploiement de TACT dans notre pays et en Afrique ?
   2. Existe-t-il des omissions de notre part mais que vous souhaiteriez mentionner ?

*Merci pour vos contributions éclairées au présent projet*
