# Supplementary material for: To what extent are the antimalarial markets in African countries ready for a transition to triple artemisinin-based combination therapies?
Source: PLoS One. 2021 Aug 31;16(8):e0256567. doi: 10.1371/journal.pone.0256567 (PMC8407563; doi:10.1371/journal.pone.0256567)
Supplement: S1 File — (ZIP) [file pone.0256567.s001.zip › Interview guides (ZIP)/5. FDG-end-users_final_French.docx]

Interview Guide 5

**Intitulé du Projet: Les Aspects éthiques, Sociaux, Règlementaires et Commerciaux du Déploiement des Thérapies à Triple Combinaison à base d’Artémisinine pour le Traitement du Paludisme en Afrique : Etudes de cas au Burkina Faso et au Nigéria**

**Groupe Cible- Utilisateurs finaux : Patients, Tuteurs et membres de la communauté**

Groupe de Discussions

*Avant de commencer le groupe de discussion, s’assurer que les participants ont reçu les informations préélaborées sur les TACT*

1. Instructions
   1. *Noter le nombre et les caractéristiques des participants aux discussions*

- ***6 -12 participants***
- ***Groupe d’âge (masculin/féminin), le niveau d’éducation/profession***
  1. *Accueillir les participants à la discussion et décrire brièvement les objectifs du projet*
  2. *Passer en revue la fiche d’informations et fournir une copie de la fiche de consentement pour signature*
  3. *Présenter le format de l’entretien (items, durée…)*
  4. *Consacrer du temps pour répondre aux questions et apporter les éclaircissements*
  5. *Demander la permission pour utiliser l’enregistreur*
  6. *S’assurer que la personne chargée de la prise des notes est prête*
  7. *Tester l’appareil d’enregistrement (possibilité d’utiliser 2 enregistreurs)*

1. Points de vue sur le Traitement et la Prévention du Paludisme
   1. Lorsque vous suspectez un accès de paludisme, quel est votre type de recours au soin ?

Quels types de médicaments utilisez-vous et pour quelles raisons ?

- 1. Que savez-vous des stratégies actuelles de lutte contre le paludisme mise en œuvre dans notre pays ?
  2. Quels sont les médicaments qui sont couramment utilisés pour traiter un accès de paludisme, selon vos connaissances ?

Quels sont les avantages et les inconvénients de ces médicaments ?

- 1. Comprenez-vous les raisons qui ont entrainé un changement du traitement antipaludique par le passage de la chloroquine aux ACT ?
  2. Quelles ont été les préoccupations des communautés lors de ce changement de protocole de traitement ? Qu’est-ce qu’on aurait pu faire pour répondre à ces préoccupations ?

1. Points de vue sur les considérations éthiques relatives au déploiement des TACT
   1. Pouvez-vous partager vos opinions sur les questions éthiques qui devraient être prises en compte avant le déploiement des nouvelles combinaisons des traitements (TACT) contre le paludisme dans notre pays ?
      1. Avis sur les risques supplémentaires pour les enfants
      2. Avis sur le fait qu’il n’y ait pas d’avantage clinique pour les patients mais que cela retardera la résistance pour les générations futures
   2. Les études ont montré les possibilités d’une croissance légère des effets secondaires comme la nausée, les vomissements mais également la capacité à prévenir la résistance aux médicaments. Qu’en pensez-vous ?

(Position par rapport au risques/malaises individuels VS avantage public)

- 1. Que pensez-vous d’un changement potentiel des ACT aux TACT comme traitement antipaludique de première ligne dans notre pays vu que les ACT y sont encore efficaces.
- Que pensez-vous de la limitation du choix des patients aux TACT seulement pour le traitement antipaludique afin de prévenir la résistance (les avantages uniquement en termes de santé public) ?
  1. Sur la base des informations qui vous sont données, pouvez-vous nous dire ce que signifie la résistance aux médicaments et la manière dont elle peut affecter la santé à venir de la communauté ?

1. Points de vue sur l’acceptabilité communautaire des TACT
   1. Quelles sont les préoccupations locales qui susciteraient l’introduction d’un nouveau traitement antipaludique dans votre communauté vue que les traitements actuels (ACT) sont toujours efficaces et constituent les traitements de première ligne ?

- (Avis sur les barrières au niveau communautaire, la compréhension du changement de protocole de prise en charge, les coûts, l’acceptabilité…)
  1. Comment devrait-on lever ces barrières ?
  2. Quelles sont les mesures qui pourraient faciliter l’acceptation des TACT

1. Points de vue sur l’accès
   1. Les traitements anti palustres sont-ils accessibles dans votre communauté ?

- Quels sont les défis actuels en matière d’accès aux traitements contre le paludisme ? (défis géographiques, financiers, physiques…)
  1. Comment pourrait-on relever ces défis ?
  2. Pouvez-vous nous expliquer la manière dont vous et votre foyer avez accès aux médicaments antipaludiques ?
  3. Pouvez-vous nous expliquer la manière dont les enfants et les femmes enceintes ont accès au traitement antipaludique dans votre communauté ?

1. Positionnement sur le marché : accessibilité
   1. Combien coûtent les médicaments antipaludiques sur le marché ? Sont-ils accessibles ?

- Quel est le lien entre le secteur public et le privé ?
  1. Quels seraient les prix au détail adéquats/acceptables pour les TACT ?

Quel serait le lien entre ces prix et ceux des ACT ?

Il y aurait-il une différence entre le secteur public et celui privé ?

- Quelles sont les activités/dispositions que le gouvernement devrait entreprendre pour rendre les TACT plus attractifs que les ACT ?
  1. Comment le coût influence-t-il le choix du traitement antipaludique des membres de votre communauté ?
  2. Quelles sont les autres considérations sur l’accessibilité que l’on devraiton prendre en compte avant que les TACT ne soient un traitement antipaludique de première ligne ?
  3. Seriez-vous disposé à acheter les TACT même si les ACT sont disponibles ?

Pourquoi ? / Pourquoi pas ?

1. Engagement et engouement de la communauté pour les TACT
   1. Comment devrait-on impliquer les communautés locales dans la discussion sur le déploiement des TACT dans notre pays ?
   2. Quelles seraient les stratégies efficaces pour faciliter le déploiement des TACT dans notre pays (Avis sur les expériences de déploiement antérieur)
   3. Sur la base de votre expérience, quelles sont les activités d’engagement communautaires qui ont été les plus efficaces? (ex Rencontres, réunions…communautaires, réunions villageoises…)
   4. Comment le déploiement des TACT influencera-t-il le comportement de recours aux soins des patient et des membres de la communauté, eu regard de la légère augmentation des effets secondaires pour les patients ?
   5. Quel type d’engagement communautaire et public (Stratégie) est nécessaire avant et pendant le déploiement des TACT ?
   6. Quelles sont les acteurs principaux et les communautés que l’on devrait cibler dans ces activités de sensibilisation / mobilisation ?
2. Positionnement sur le marché : Sélection du produit
   1. Selon vous, qui décide-t-il des types des traitements antipaludiques à utiliser dans notre pays ?

Dans quelle mesure le consommateur a-t-il son mot à dire ?

- 1. Personnellement, comment décidez-vous des traitements contre le paludisme que vous achetez ? (ex prix, marque, effets secondaires, directives, accessibilité…)
  2. Quel type d’information sur la résistance exigeriez-vous pour vous engager dans l’adoption des TACT ? Comment ces informations devraient-elles être fournies ?
  3. Qu’elles sont, selon vous, les activités promotionnelles adéquates pour informer le public du changement de médicaments de première ligne pour les TACT ?
     (instructions imprimées, filigrane sur les boîtes…)
  4. Selon vous, quelles sont les autres aspects commerciaux à prendre en compte pour faire face au changement des ACT aux TACT ?

1. Positionnement du marché : Régime de traitement
   1. Comment suivez-vous le régime de traitement aux ACT ? Arrivez-vous a respectez les instructions pour la prise des médicaments ?
   2. Nous prévoyons que le nombre de comprimés soit similaire à celui des ACT actuels, toutefois en cas d’augmentation, quel nombre serait acceptable ?

- Quelles sont vos considérations relatives à l’adhésion au suivi complet du traitement ACT ?
  1. L’ajout d’une troisième composante peut entrainer des effets secondaires légers. Par exemple, entrainer plus de vomissements chez des patients dans l’heure suivant le traitement (1 sur 100 pour les ACT contre 3 sur 100 pour les TACT).
     Pensez-vous que cela est acceptable ?
- Qu’en serait-il des autres effets secondaires comme la fatigue, les vertiges, les maux de tête etc. qui pourraient s’accentuer avec les TACT comparé aux ACT ?
  1. Quelles sont les autres facteurs que l’on devrait prendre en compte concernant l’acceptation des TACT ?

1. Recommandations
   1. En se basant sur nos échanges, quelles recommandations feriez-vous pour relever les principaux défis et briser les barrières au déploiement des TACT dans votre communauté en particulier ? Dans notre pays en général ?
   2. Existe-t-il des omissions de notre part mais que vous souhaiteriez mentionner ?

*Merci pour vos contributions éclairées au présent projet*
