# Supplementary material for: To what extent are the antimalarial markets in African countries ready for a transition to triple artemisinin-based combination therapies?
Source: PLoS One. 2021 Aug 31;16(8):e0256567. doi: 10.1371/journal.pone.0256567 (PMC8407563; doi:10.1371/journal.pone.0256567)
Supplement: S1 File — (ZIP) [file pone.0256567.s001.zip › Interview guides (ZIP)/5. FGD-enduser_Final_English.docx]

Interview Guide 5

**Project Title: Ethical, Social, Regulatory and Market related aspects of Deploying Triple Artemisinin-Based Combination Therapies for Malaria treatment in Africa: Case studies in Burkina Faso and Nigeria**

Target group-End Users: Patients, Guardians and Community members

Focus Group Discussions

*Before starting the FGD, make sure /verify that participants received the pre-developed information about TACT*

1. Instructions
   1. *Note number and characteristics of discussants*

- ***6-12 discussants***
- *Age group, (male/female), level of education / profession*
  1. *Welcome the discussants and briefly describe objectives of the project*
  2. *Review Study Info Sheet & provide copy of Consent Form for signature*
  3. *Allow time for questions and clarifications*
  4. *Request for permission to use audio-recorder*
  5. *Outline the format of interview*
  6. *Ensure note-taker is ready*
  7. *Test audio-recorder (possibly use 2 recorders)*

1. General views on Malaria Control and Prevention
   1. What is your treatment seeking behavior in case you expect an episode of malaria? What kind of medications do you use and why?
   2. What do you know about the current strategies for malaria control?
   3. What medicines are commonly used to treat a malaria infection? What are the advantages and disadvantages of these medicines?
   4. Do you understand the reasons for a change in malaria treatment from Chloroquine to ACTs?
   5. What were some of the community concerns around that change of treatment policy? What could have been done to address these local concerns?
2. Views on key ethical considerations on deployment of TACTs
   1. Could you please share your views on what ethical issues should be considered before the deployment of new combinations of antimalarials in this country?
      - Probe for additional risks for children
      - Probe for no clinical benefit to patients but delay in multidrug resistance for future generations
   2. Studies have shown the possibilities of slight increase in minor side effects such as nausea, vomiting) but could prevent antimalarial drug resistance. What are your views? (Probe for individual risks/discomforts vs public benefit)
   3. What are your views on a potential change from ACTs to TACTs as a first line treatment for malaria in your country, given that ACTs are still effective in this country?

- What are your views on limiting patients’ choice to just TACTs for malaria treatments to prevent resistance (for public health benefits)?
  1. From the information just given to you, can you recap to me what drug resistant means and how it can affect the future health of our community?

1. Views on community acceptability of TACTs
   1. What are the local concerns that would arise in the introduction of a new malarial treatment in this community, given that current treatments (ACTs) are still effective and currently the first line of treatment?

- (Probe for community level barriers, understanding of the change in policy, costs, acceptability)
  1. In what key ways should these barriers be addressed?
  2. What measures could facilitate community acceptance of TACTs

1. Views on accessibility
   1. How accessible are malaria treatments in this community?

- What are the current challenges in getting access to malaria treatments (probe for geographic, financial, physical challenges)?
  1. In what key ways should these challenges be addressed?
  2. Can you explain to me how you and your household currently access anti-malaria drugs?
  3. Can you explain to me how children and pregnant women access anti-malaria drug in your community?

1. Market positioning: affordability
   1. How costly are anti-malarial drugs in the market? Is it affordable?

- How does this differ between the public and private sector?
  1. It is likely that TACT might be slightly more expensive than ACT. What would be appropriate/acceptable retail prices for TACT? How would these have to relate to ACT? What activities could the government take to make TACT more attractive than ACT?
  2. In what ways does price affect the choice of malaria treatment for members of this community.
  3. What other considerations regarding affordability should be made before TACT can become first-line anti-malarial?
  4. Would you be prepared to buy TACT, even when ACT is available? Why (not?)

1. Community engagement and uptake of TACTs
   1. In what key ways should local communities be engaged in discussions on deployment of TACTs in this country?
   2. What would be effective strategies to facilitate the deployment of TACTs in this country? (Probe for experiences from previous deployment)
   3. In your experience, what are some of the community engagement activities that have been most effective (probe for community meetings, village durbars etc.)
   4. How will the deployment of TACTs influence the health seeking behavior of patients and community members, given the slight increase in minor side effects for patients?
   5. What type (strategies) of community and public engagement is necessary prior to and during deployment?
   6. Which key communities and stakeholders should be targeted in these engagement activities?
2. Market positioning: product selection
   1. According to you, who decides which anti-malarial medicines are being used in the country? To what extent does the consumer have a saying in this?
   2. Personally, how do you decide what anti-malarial medicines you buy (e.g. price, brand, side-effects, guidelines, availability)?
   3. What type of information about resistance would you require to engage in adopting TACT? How should this information has to be provided?
   4. What promotional activities are deemed appropriate to inform the public of changing first-line drugs to TACT (eg. printed instructions, watermarks on box)?
   5. What other market related issues do you think would be necessary to address for a switch from ACT to TACT?
3. Market positioning: treatment regime
   1. How well do you comply with the drug treatment regimen of anti-malarials? For example the full amount of pills during three days
   2. We expect the number of tablets for TACT treatment to be similar to the existing ACT, but if there is an increase in the number of tablets, what would be acceptable?

- What are your considerations with regard to adherence to the full treatment regime of TACT?
  1. The addition of a third component may have some slight side effects. For example, adding a third drug can results in more patients vomiting within one hour of treatment (1 per 100 for ACT, versus 3 in 100 for TACT). Would this be acceptable?
- How would this be for other side-effects such as fatigue, dizziness, headache etc, that might slightly increase with TACT compared to ACT?
  1. What other factors should be taken in consideration with regard to acceptance of TACT?

1. Recommendations
   1. Based on our discussions, what recommendations would you give for addressing the key challenges and barriers to deploying TACTs in your community/country?
   2. Is there anything that we haven’t covered that you’d like to mention?

*Thank you very much for your insightful inputs to this project*
