# Supplementary material for: To what extent are the antimalarial markets in African countries ready for a transition to triple artemisinin-based combination therapies?
Source: PLoS One. 2021 Aug 31;16(8):e0256567. doi: 10.1371/journal.pone.0256567 (PMC8407563; doi:10.1371/journal.pone.0256567)
Supplement: Attachment — Submitted filename: renamed_0870f.docx [file pone.0256567.s002.docx]

Reviewer’s comments:

The authors have explored the extent to which the antimalarial drug markets in African countries are ready for a transition to TACTs piloting with Nigeria and Burkina Faso. This is posited in the African setting where ACTs are still effective and TACTs would not additionally benefit the individual patient but rather future patients and the larger community by delaying or preventing artemisinin and partner drug resistance. They show that market prospects of TACTs in Nigeria and Burkina Faso will depend on the demonstration of the added value over current ACTs, their inclusion in financial arrangements, and the alignment of TACTs with current distribution and deployment practices. They also establish the need for further clinical, health economic and feasibility studies to inform decision makers about the broader implications of a transition to TACTs. They also highlight the recent emergence of ACT resistance in some African Countries and reduced efficacy, as important determinants of the market readiness for TACTs.

The manuscript is concisely written, well structured and provides adequate and important content to address the subject.

I suggest that the authors should include the questionnaire used for the interviews as supplementary data.

.
